# Prevalence and risk factors of axial neck pain in patients undergoing multilevel anterior cervical decompression with fusion surgery

**DOI:** 10.1186/s13018-019-1132-y

**Published:** 2019-04-04

**Authors:** Sen Liu, Da-Long Yang, Ruo-Yu Zhao, Si-Dong Yang, Lei Ma, Hui Wang, Wen-Yuan Ding

**Affiliations:** 1 Department of Spinal Surgery, The Third Hospital of Hebei Medical University, 139 Ziqiang Road, Shijiazhuang, 050051 People’s Republic of China; 2 Hebei Provincial Key Laboratory of Orthopaedic Biomechanics, 139 Ziqiang Road, Shijiazhuang, 050051 People’s Republic of China

**Keywords:** Risk factor, Axial neck pain, Kyphosis, Multilevel anterior cervical decompression with fusion

## Abstract

**Objectives:**

The aim of this study was to explore the prevalence and risk factors for axial neck pain in patients undergoing multilevel anterior cervical decompression with fusion surgery.

**Methods:**

In this study, 88 patients, who underwent multilevel anterior cervical decompression with fusion surgery from January 2012 to January 2017, were retrospectively reviewed. Based on the postoperative axial neck pain, the patients were classified into two groups: axial pain group and no axial pain group. The patients were followed up 3 weeks, 3 months, and 1 year after cervical anterior surgery for the early- and long-term clinical evaluation. The possible effect factors included demographic variables (age, sex, BMI, smoking, drinking, heart disease, hypertension, diabetes, preoperative kyphosis, preoperative axial neck pain, preoperative JOA scores, and ODI) and surgery-related variables (surgical option, vertebral lesions, spinal canal stenosis rate, superior fusion segment, presence of intramedullary high signal intensity).

**Results:**

The prevalence of axial neck pain was 27.3% (24 cases of 88). Our results showed that preoperative axial neck pain (62% vs 23%, *P* < 0.001) and preoperative kyphosis (42% vs 21.9%, *P* < 0.001) were risk factors for axial pain after multilevel anterior cervical surgery. Additionally, for patients with preoperative cervical kyphosis, compared to no axial pain group, the axial neck group was significantly more likely to exist a higher preoperative angle of C2–7 (13.31 ± 2.33 vs 7.33 ± 2.56, *P* < 0.001) and a higher correction range for kyphosis (20.24 ± 4.12 vs 12.34 ± 3.12, *P* < 0.001). However, for all the patients with postoperative axial symptoms, the improvement rate of axial pain was significantly higher for patients without cervical kyphosis at the early-term follow-up (3 weeks) (*P* = 0.032), no significant differences were found at the medium-term (*P* = 0.554) and long-term follow-up (*P* = 0.902), and improvements of clinical symptom have no obvious difference at the last follow-up.

**Conclusions:**

Overall, preoperative axial neck pain and kyphosis could predict axial neck pain for patients undergoing multilevel anterior cervical decompression with fusion surgery, and recovery of cervical kyphosis may contribute to the long-term recovery of neural function, but may also suffer from risk of short-term axial pain, which could be reduced through moderate cervical curvature recovery.

## Introduction

Cervical spondylotic myelopathy (CSM) is a common clinical degenerative disease with an incidence of about 53.5% [1, 2], seriously impacting quality of life and even causing disability for the elderly population [3, 4], which can also lead to defecation dysfunction even paralysis if accompanied by cervical cord injury. For patients with multilevel CSM, anterior decompression and instrumentation, including anterior cervical corpectomy and fusion (ACCF), anterior cervical discectomy and fusion (ACDF), anterior cervical hybrid decompression and fusion (ACHDF), have grown in popularity due to improvement in technology and surgical skill that allows direct decompression and reconstruction with satisfied outcome [5–8]. However, in many cases, anterior cervical decompression with fusion surgery is still associated with unresolved complications, including dysphagia, postoperative hematoma (neck), hoarseness, esophageal injury, injury to major vessels, wound infection (neck), graft extrusion, axial neck pain, C5 palsy, reduction in neck motion, pseudoarthrosis, nonunion, and revision and screw removal [9, 10]. Among them, axial neck pain as a common complication after surgery, especially in patients undergoing multilevel anterior or posterior cervical decompression, severely threatens the physical and mental health and life quality of the patients. Axial pain has been defined as a chronic, dull ache extending from the nuchal to the periscapular or shoulder region with the feeling of acid bilges, stiffness, oppression, and muscle spasm and is diagnosed after eliminating diseases associated with other related systems [11–15]. Multilevel anterior decompression and instrumentation often needs to insert intervertebral graft, not only for fusion but also for reconstruction of intervertebral height to indirectly decompress by distraction. Previous literature has reported postoperative neck pain might come out of overdistraction by inserting a large instrumentation, which was considered to lead to posterior facet joint distraction or posterior neck muscle spasm. However, no evidence that supports the relationship between graft size and postoperative axial neck pain has been available. Persistent axial pain can be a major cause of dissatisfaction after surgery, even in patients with excellent neurological recovery. With the emerging appreciation of health-related quality of life, the symptom of pain merits attention by the virtue of it being commonly linked to ongoing disease in the mindset of patients. The incidence of axial pain in individuals with posterior cervical decompression is reportedly as high as 60–80% according to previous articles [16, 17]. Although axial pain has gradually been receiving more attention, compared with a monumental amount of coverage of posterior decompression, this complication associated with multilevel anterior cervical decompression is seldom described in large clinical series [18, 19]. The aim of this study was to explore prevalence and risk factors for axial neck pain in patients undergoing multilevel anterior cervical decompression with fusion surgery.

## Materials and methods

### Inclusion of patients

Between January 2012 and January 2017, a total of 88 consecutive patients (45 men and 43 women) who were diagnosed with multilevel CSM and needed operative managements were examined prospectively. The Ethics Committee of The Third Hospital of Hebei Medical University approved the study, and written informed consents were obtained from all patients before they were recorded. The inclusion criteria were the following: multilevel cervical spondylotic myelopathy of cervical spinal stenosis with neurological dysfunctions; inefficacious patients treated with conservative treatment; and 3- and 4-level anterior cervical decompression and instrumentation including ACCF (2-level corpectomy), ACDF (3- or 4-level), ACHDF (1-level corpectomy plus 1- or 2-level discectomy). The exclusion criteria were the following: the presence of ossification of the posterior longitudinal ligament; the presence of infection and trauma; cervical ossification of the ligamenta flava; association with thoracic or lumbar diseases or spine deformity; previous history of spinal surgery; and unwillingness to participate in the study.

### Study variables

The possible risk factors include two parts: demographic variables and surgical-related variables. The following are the demographic variables collected at baseline: age, sex, BMI, smoking, drinking, heart disease, hypertension, diabetes, preoperative kyphosis, preoperative axial neck pain, preoperative JOA scores, and ODI. And the surgical-related variables include the following: surgical option, lesions vertebral, spinal canal stenosis rate, superior fusion segment, and presence of intramedullary high signal intensity.

**Fig. 1:**
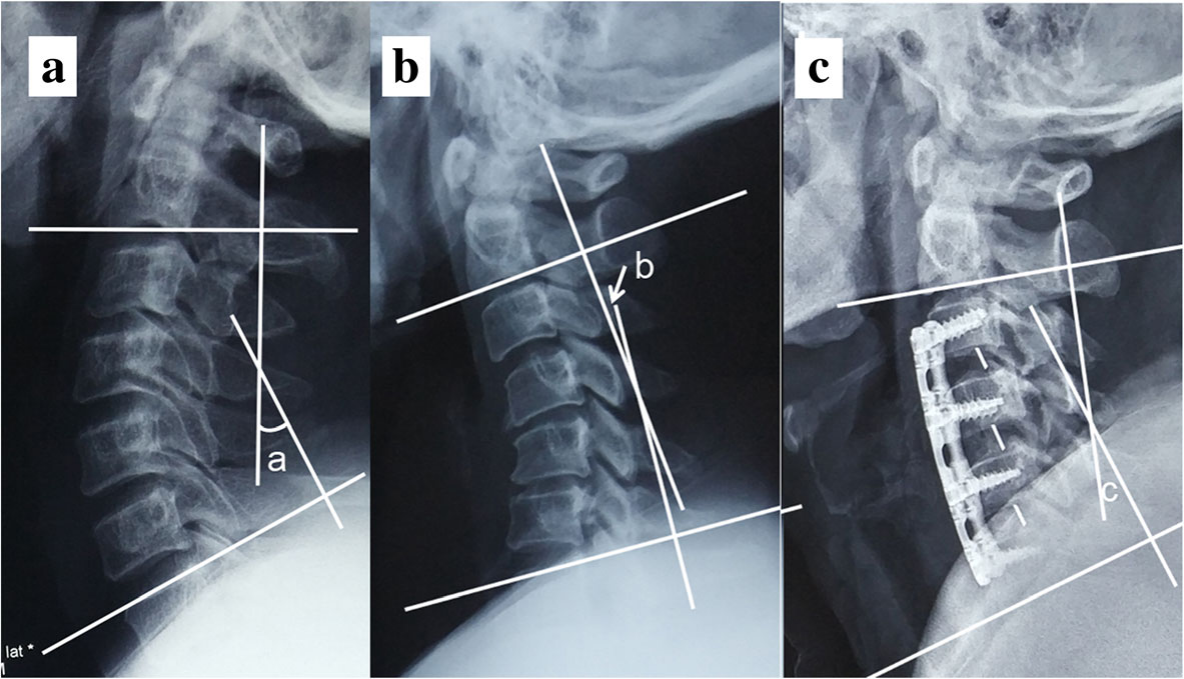
The cervical lordosis (CL), namely as C2–7 Cobb angle, was calculated by measuring the angle between the C2 subvertebral endplate plane and the extension line of C7 subvertebral endplate plane at preoperation (**A**, **B**) and postoperation (**C**) on lateral radiographs. Anterior convex was positive (a) and posterior convex was negative (b)

All the patients underwent cervical neutral lateral X-ray film, and the cervical lordosis (CL), namely as C2–7 Cobb angle, was calculated by measuring the angle between the C2 subvertebral endplate plane and the extension line of C7 subvertebral endplate plane at preoperation and postoperation on lateral radiographs. Anterior convex was positive and posterior convex was negative (Fig. 1).

### Axial neck pain evaluation

The visual analog scale (VAS) is a sensitive and reliable clinical procedure for the assessment of pain degree, which consists of a horizontal line 100 mm in length. The ends of the horizontal line point “0” and “100,” respectively representing “no pain” and “worst imaginable pain.” The middle section shows different degrees of pain [20]. Based on the postoperative axial neck pain, the patients were classified into two groups: the axial pain group, including patients with obvious pain and related pain treatment, and the no axial pain group, including patients with no axial pain or with slight discomfort and without treatment. The patients were followed up 1 year after cervical anterior surgery, and the medium- and long-term clinical evaluations were respectively collected. We chose the 1-year follow-up interval, the time we believe outcomes were expected to be optimal, to assess the clinical efficacy of the patients.

### Statistical analysis

Comparative analysis with postoperative axial neck pain as the dependent variable was done using independent samples *t* tests and chi-square. Age, BMI, preoperative axial neck pain, lesions vertebral, spinal canal stenosis rate, preoperative JOA scores, and ODI were analyzed using independent samples *t* tests, and sex, smoking, drinking, heart disease, hypertension, diabetes, preoperative kyphosis, preoperative axial neck pain, surgical option, lesions vertebral, superior fusion segment, and presence of intramedullary high signal intensity were analyzed using chi-square. The statistical significant value was set at *P* < 0.05 in the univariate analyses. All statistical analyses were carried out by SPSS software version 13.0 (SPSS, Inc., Chicago, IL, USA).

## Results

On the day before surgery, 88 patients (45 men and 43 women) were registered on the books for evaluation voluntarily. The cohort of patients was integrated before discharge. At the time of 1-year follow-up, no patient was lost to follow-up. Thus, 88 patients (45 men and 43 women) had entered into the final assessment phase. Based on the postoperative axial neck pain, the patients were classified into two groups: axial pain group, including patients with obvious pain and related pain treatment, and no axial pain group, including patients with no axial pain or with slight discomfort and without treatment.

**Fig. 2:**
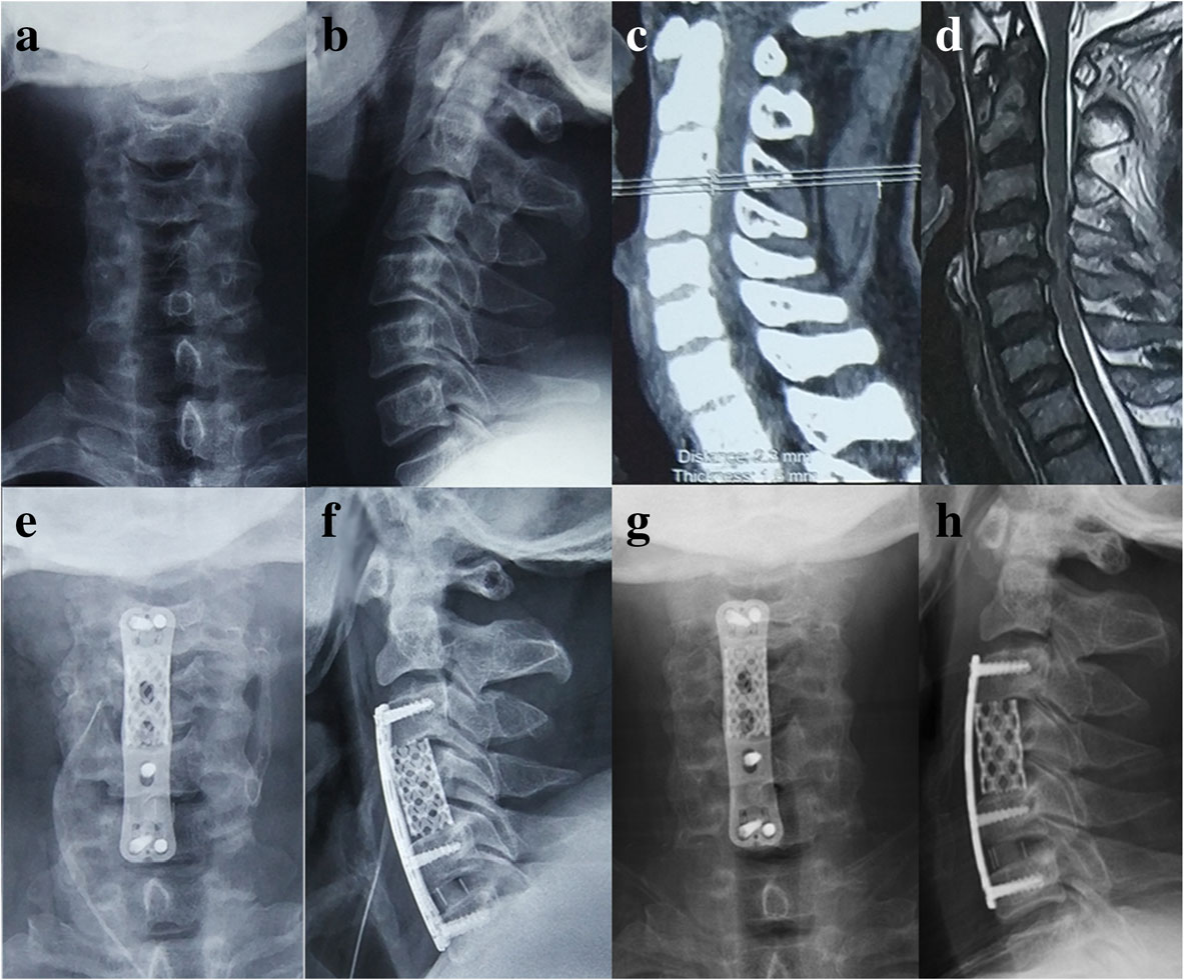
A 52-year-old male developed numbness and weakness in his four extremities for 2 years, together with unbalance gait for 2 months. Preoperative radiographs showed that the sagittal alignment of the cervical spine was physiologic lordosis (**a**, **b**), and the magnetic resonance imaging scans showed that the spinal cord compressed at C3/4, C4/5, C5/6 (**c**, **d**). He was performed with ACHDF including 1-level corpectomy plus 1-level discectomy without surgery-related complications. After operation, his JOA scores improved from 9.7 preoperation to 13.6 postoperation. Postoperative lateral and flexion-extension cervical radiographs showed that the cervical kyphosis was corrected (**e**, **f**) and the graft was fused at 1-year follow-up (**g**, **h**)

**Fig. 3:**
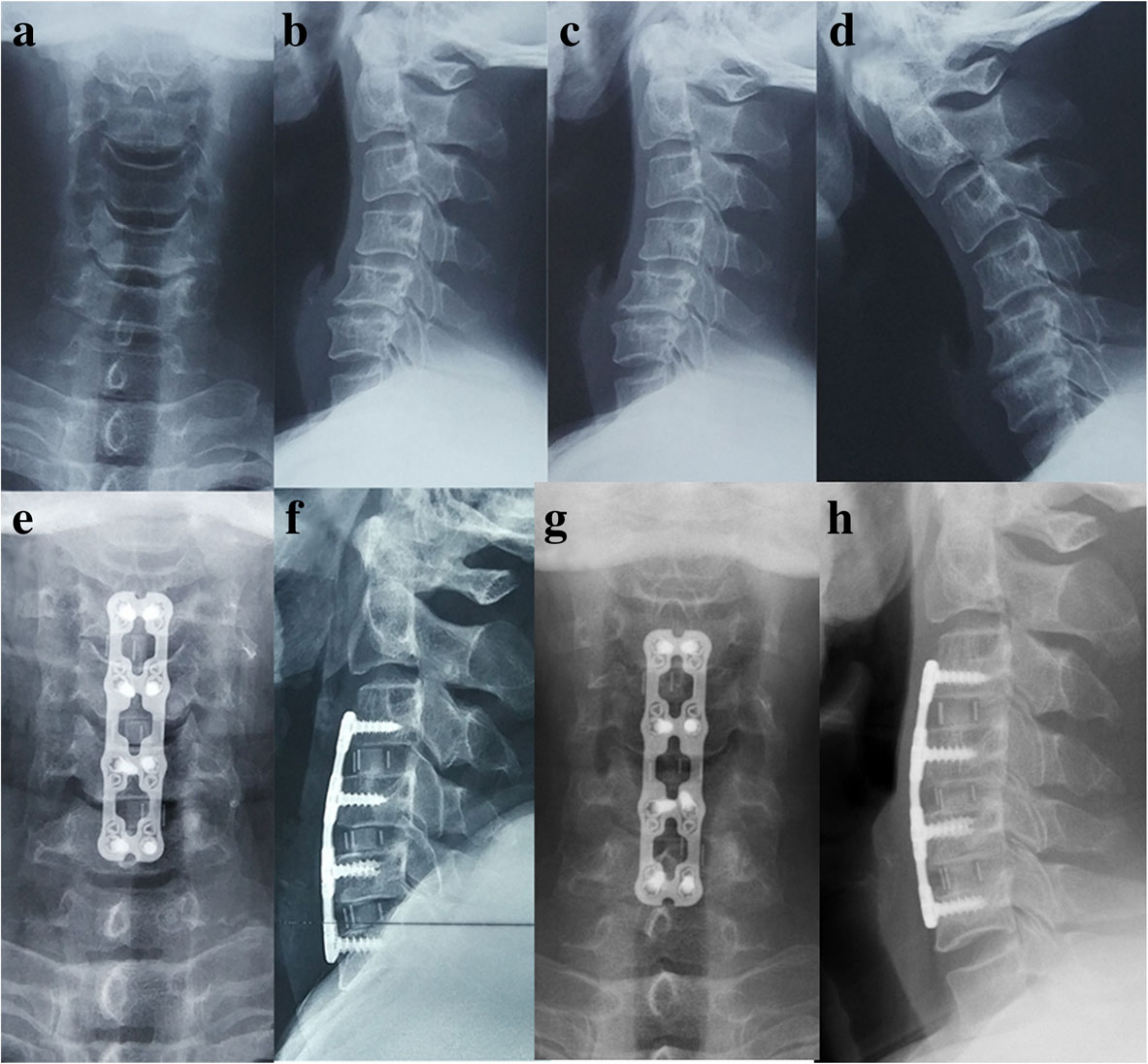
A 60-year-old male developed numbness in his two hands and weakness in his four extremities for 3 years. Preoperative radiographs showed that the sagittal alignment of the cervical spine was kyphotic (**a**–**d**). He was performed with 3-level ACDF and presented with axial neck pain without other surgery-related complications. After operation, his JOA scores improved from 9.4 preoperation to 14.7 postoperation and axial neck pain disappeared 2 months after surgery. Postoperative lateral and flexion-extension cervical radiographs showed that the cervical lordosis was improved (**e**, **f**) and the graft got bony fusion at 1-year follow-up (**g**, **h**)

There were 24 patients in the axial pain group and 64 patients in the no axial pain group, and the prevalence of axial neck pain was 27.3%. According to the statistical analyses of demographic variables, preoperative axial neck pain (62% vs 23%, *P* < 0.001) and preoperative kyphosis (42% vs 21.9%, *P* < 0.001) had a significant difference between the two groups (Figs. 2 and 3). However, no significant differences were found in age (59.5 ± 8.8 vs 60.7 ± 9.8, *P* = 0.600), sex (*P* = 0.542), body mass index (BMI) (25.3 ± 2.9 vs 23.8 ± 4.9, 0.143), smoking (*P* = 0.430), drinking (*P* = 0.219), heart disease (*P* = 0.580), hypertension (*P* = 0.551), diabetes (*P* = 0.683), JOA scores (9.95 ± 2.1 vs 9.98 ± 1.53, 0.248), DOI scores (0.52 ± 0.08 vs 0.54 ± 0.11, 0.378) **(**Table 1).

**Table 1:** The main demographic variables of patients before the surgery

	Axial pain (*n* = 24)	No axial pain (*n* = 64)	*P* value
Age (years)	59.5 ± 8.8	60.7 ± 9.8	0.600
Sex (male/female)	11/13	34/30	0.542
BMI (kg/m^2^)	25.3 ± 2.9	23.8 ± 4.9	0.143
Smoking (yes/no)	9/15	30/34	0.430
Drink (yes/no)	6/18	25/39	0.219
Heart disease	7/17	15/49	0.580
Hypertension (yes/no)	7/17	23/41	0.551
Diabetes (yes/no)	5/19	16/48	0.683
Preoperative kyphosis	11/13	14/50	0.026
Axial neck pain	15/9	18/46	0.003
JOA scores	9.95 ± 2.1	9.98 ± 1.53	0.248
DOI scores	0.52 ± 0.08	0.54 ± 0.11	0.378

There were statistically significant differences between preoperative kyphosis and axial neck pain in two groups (*P* < 0.05)

According to the statistical analyses of surgical-related variables, no significant differences were found on the following factors: course of disease (11.03 ± 2.45 vs 11.98 ± 4.13, *P* = 0.294), operation time (96.9 ± 16.5 vs 103.1 ± 30.6, *P* = 0.348), surgical option (*P* = 0.187), superior fusion segment (*P* = 0.499), incision length (8.87 ± 0.87 vs 9.25 ± 1.19, *P* = 0.156), blood loss (253.5 ± 19.2 vs 266.3 ± 30.0, *P* = 0.055), preoperative VAS-neck (4.35 ± 1.13 vs 4.00 ± 0.92, *P* = 0.069), and presence of IHSI on MRI (*P* = 0.563) (Table 2).

**Table 2:** The surgery-related variables of patients

	Axial pain (*n* = 24)	No axial pain (*n* = 64)	*P* value
Course of disease (months)	11.03 ± 2.45	11.98 ± 4.13	0.294
Operation time (min)	96.9 ± 16.5	103.1 ± 30.6	0.348
Surgical option			0.187
ACDF	9	38	
ACCF	1	2	
ACCDF	14	24	
Superior fusion segment			0.499
C3–6	15	34	
C4–7	8	29	
C3–7	1	1	
Incision length (cm)	8.87 ± 0.87	9.25 ± 1.19	0.156
Blood loss (ml)	253.5 ± 19.2	266.3 ± 30.0	0.055
Presence of IHSI on MRI (yes/no)	5/19	10/54	0.563

There were no statistically significant differences between the surgery-related variables in the two groups (*P >* 0.05)

Additionally, for patients with preoperative cervical kyphosis, compared to the no axial pain group, the axial neck group was significantly more likely to exist a higher preoperative angle of C2–C7 (13.31 ± 2.33 vs 7.33 ± 2.56, *P* < 0.001) and a higher correction range for kyphosis (20.24 ± 4.12 vs 12.34 ± 3.12, *P* < 0.001) (Table 3). However, for all the patients with postoperative axial symptoms (*n* = 24), the improvement rate of axial pain was significantly higher for patients without cervical kyphosis at the early-term follow-up (3 weeks) (*P* = 0.032), and no significant differences were found at the medium-term (*P* = 0.554) and long-term follow-up (*P* = 0.902) (Table 4).

**Table 3:** Comparison of C2–7 Cobb angle between the patients presenting preoperative kyphosis in the two groups

	Preoperative kyphosis		*P* value
	Axial pain (*n* = 11)	No axial pain (*n* = 14)	
Angle of C2–7 (°)	− 12.65 ± 3.09	− 7.05 ± 1.64	< 0.001
Correction range for kyphosis (°)	20.07 ± 3.99	12.57 ± 3.65	< 0.001

For all the patients with preoperative cervical kyphosis, the axial neck group was significantly more likely to exist a higher preoperative angle of C2–7 (*P* < 0.001) and a higher correction range for kyphosis (*P* < 0.001)

**Table 4:** Effects of preoperative kyphosis on clinical outcomes of postoperative axial pain

	Postoperative axial pain(*n* = 24)		*P* value
	Kyphosis (*n* = 11)	No kyphosis (*n* = 13)	
Axial pain after surgery (yes/no)			
3 weeks	9/2	5/8	0.032
3 months	4/7	3/9	0.554
12 months	1/10	1/12	0.902
JOA scores 1 year later	14.88 ± 1.46	14.07 ± 1.45	0.187

For all the patients with postoperative axial pain, the improvement rate of axial pain was significantly higher for patients without cervical kyphosis at the early-term follow-up (3 weeks) (*P* < 0.05), and no significant differences were found at the medium-term and long-term follow-up

## Discussion

Degenerative spine diseases such as CSM are increasing among the geriatric population, and surgical treatment of CSM is becoming more common. Consensus has currently been reached on the surgical management of CSM involving one or two mobile segments; however, controversy remains regarding the selection of surgical procedures for treatment of multilevel CSM, especially 3- or 4-level CSM [21–25]. Li et al. [26] reported that anterior techniques had become one of the most popular spinal surgeries for the treatment of CSM, which not only allowed direct decompression, but could also help restore the height of interbody spaces and restore cervical lordosis with careful intraoperative distraction, and the immediate stability of the cervical spine could be achieved with grafting and with anterior internal fixation. Axial neck pain has been recognized as one of the most important complications after cervical surgery. The incidence of axial pain in individuals with posterior cervical decompression is reportedly as high as 60–80% according to previous articles [16]. Kawaguchi et al. reported that postoperative axial pain for posterior cervical decompression was associated with the destruction of posterior cervical muscle complex and abnormal cervical curvature [17]. For patients undergoing anterior cervical decompression surgery, previous researches have investigated detailed complications, such as dysphagia, postoperative hematoma (neck), hoarseness, esophageal injury, injury to major vessels, wound infection (neck), graft extrusion, axial neck pain, C5 palsy, reduction in neck motion, pseudoarthrosis, nonunion, and revision and screw removal. To the best of our knowledge, however, few studies focus on the axial neck pain associated with anterior decompression, especially multilevel decompression with fusion surgery.

In this study, radiologic images of the patients showed that spinal cord compressions in the majority of multilevel CSM were mainly the result of protrusive intervertebral discs and osteophytes, and the pathophysiologic features of multilevel CSM make anterior decompression the most effective surgical option. We found that the prevalence of axial neck pain was 27.3% for patients undergoing multilevel anterior cervical decompression with fusion surgery, which was significantly lower than that of posterior decompression. This might suggest that the destruction of posterior cervical muscle complex had a significant effect on postoperative axial symptoms. On the basis of perioperative clinical and radiographic parameters, we compared three different anterior techniques, ACCF, ACDF, and ACHDF, for the treatment of 3- and 4-level CSM. The result shows that different surgical procedures for postoperative axial pain make no difference. The key finding of this study is that preoperative axial neck pain and kyphosis are the risk factors of axial neck pain for patients undergoing multilevel anterior cervical decompression with fusion surgery, and moderate correction of the kyphosis is more significant to avoid the axial pain.

The physiological curvature of the cervical vertebrae is an arcuate protuberance in the middle of the cervical segment of the human spine, which evolved over a long period of time to adapt to the upright walking posture of the human body. Lee et al. [27] pointed out that the normal value of cervical curvature in the middle position was 12~33°, and the C2–7 Cobb angle of cervical spine was abnormal in males with less than 20° and in females with less than 12°. In this study, the physiological curvature of cervical vertebrae was observed by preoperative and postoperative X-ray, on which the changes were obvious and easy to measure and could run through the whole process of cervical spine disease. The maintenance of normal cervical curvature includes static stability factors and dynamic stability factors. The former includes vertebral sequence, upper and lower facet joints, articular capsule, intervertebral disc, intervertebral ligament, and so on. The latter includes muscle group and ligaments around the cervical vertebrae. Dulor et al. [28] in animal studies found that the skeletal muscle led to denervated muscle during degeneration, causing muscle atrophy or replaced by adipose tissue. Previous research reported that changes in cervical curvature were accompanied by significant paraspinal muscle degeneration (fat infiltration). Thakar et al. [29] found that 93.50% of the patients with cervical spondylosis had abnormal cervical curvature, and in the control group without cervical spondylosis, only 38.50% had abnormal cervical curvature, which was significantly statistically different between the two groups. It can be seen that the changes of physiological curvature of cervical vertebrae can, to some extent, reflect the process of cervical degeneration. For patients with reversed cervical curvature, large interbody fusion cages are often needed to facilitate the recovery of cervical kyphosis. In the present study, cervical lordosis of fusion segments was significantly increased in all the patients with preoperative cervical kyphosis, but the correction range for kyphosis was greater in the axial pain group than that in the no axial pain group. Chang et al. [30] reported that a significant relationship between the increases in intervertebral disc height and interfacet distance, indicating that a large graft material lead to an increase in interfacet distance. Anterior approach allows the surgeon to distract and restore disc height, which can correct the in-buckling of the ligamentum flavum and restore alignment. It may be that multilevel ACDF can restore alignment by pulling the involved vertebral bodies toward the lordotic ventral plate, but long corpectomy grafts may straighten the cervical spinal column between the remaining vertebral bodies. Bogduk reported that provocative injection in the facet joint led to posterior neck and shoulder pains in asymptomatic volunteers [31]. The mechanism of action of pain provocation by the facet joint was suggested to be via the entrapment of synovial villi, nerve impingement by osteophyte, release of inflammatory mediators, and stretching of the facet joint capsule [32, 33]. Larger interbody fusion cages can reconstruct intervertebral space height to obtain higher cervical curvature recovery; however, overdistraction by inserting a large graft material was generally considered to lead to postoperative neck pain due to distraction of the posterior facet joint or spasm of the posterior neck muscle. We also found that for all the patients with postoperative axial neck, the absence of the axial pain was maintained at the 3-week follow-up, and improvements of clinical symptom have no obvious difference at the last follow-up. Axial symptoms gradually decrease or even disappear after a long period of adaptation. Thus, moderate cervical curvature recovery can avoid the occurrence of posterior cervical axial symptoms. Recovery of cervical kyphosis may contribute to the recovery of neural function, but may also suffer from short-term axial pain. And the exact critical values of height of intervertebral space and cervical curvature that cause postoperative axial symptoms still need further study.

This study is associated with several limitations. First, due to its retrospective design, our results did not rule out or compensate the diverse possible causes of postoperative neck pain. Therefore, further study is needed to prospectively evaluate the nature of postoperative neck pain after multilevel anterior cervical decompression with fusion surgery according to the fusion level and the nature and location of neck pain. Second, different people may have different subjective feelings about the same thing because of the different environment. In our study, only a part of all variables were selected to study which may lead to exist selection bias. Third, the effect of other postoperative complications on the postoperative neck pain was not taken into account.

## Conclusion

Overall, preoperative axial neck pain and kyphosis could predict axial neck pain for patients undergoing multilevel anterior cervical decompression with fusion surgery, and recovery of cervical kyphosis may contribute to the long-term recovery of neural function, but may also suffer from risk of short-term axial pain, which could be reduced through moderate cervical curvature recovery.
